# Recurrent Obturator Hernia: A Rare Entity

**DOI:** 10.7759/cureus.53732

**Published:** 2024-02-06

**Authors:** Jessica Biller, Jennifer Silvis, D'Arcy Duke

**Affiliations:** 1 General Surgery, Conemaugh Memorial Medical Center, Johnstown, USA; 2 Trauma and Acute Care Surgery, Conemaugh Memorial Medical Center, Johnstown, USA; 3 Bariatric and Minimally Invasive Surgery, Conemaugh Memorial Medical Center, Johnstown, USA

**Keywords:** small bowel obstruction, recurrent hernia, acute care surgery, obturator hernia, minimally invasive surgery

## Abstract

Obturator hernias are rare with an incidence of less than 1% of all hernias and are most common in frail, elderly females. They are difficult to diagnose and even more difficult to repair. They often present with a small bowel obstruction from the incarcerated bowel. We report a case of a recurrent obturator hernia after a laparoscopic repair using a patch of omentum. The recurrence was repaired laparoscopically with a trans-abdominal preperitoneal repair (TAPP) with mesh. Given the rarity of the disease, there is scarce literature on the ideal method of repair, especially in patients with recurrence. However, with recent trends toward minimally invasive preperitoneal mesh hernia repairs for inguinal and ventral hernias, this type of repair should be strongly considered for patients with obturator hernias as well.

## Introduction

Obturator hernias are rare and typically occur in thin, multiparous, elderly females due to increased pelvic laxity and malnutrition [1,2]. Additional risk factors include chronic constipation, chronic obstructive pulmonary disease (COPD), ascites, and kyphoscoliosis due to increased intra-abdominal pressure [2]. They are more common on the right side due to the protective effect of the sigmoid colon on the left, and often present with small bowel obstruction (SBO) without a palpable inguinal bulge [1]. Approximately 15%-20% of patients may present with pain in the anteromedial thigh, worsened by internal rotation of the ipsilateral hip, known as Howship-Romberg sign [1,3]. These hernias are difficult to diagnose solely by physical exam and often require computed tomography (CT). Due to the potential for delayed diagnosis, they are associated with a high risk for mortality if they contain strangulated bowel [3]. Once found, they require emergent surgical intervention to reduce the risk of bowel ischemia. The preferred management options have been debated due to their rarity. Surgical repair options range from open or laparoscopic primary suture repair, herniorrhaphy with uterus or bladder, or trans-abdominal preperitoneal mesh repair (TAPP) [1]. Here, we present a patient who initially underwent laparoscopic primary repair with omental herniorrhaphy which recurred within four years requiring reoperation.

## Case presentation

**Figure 1:**
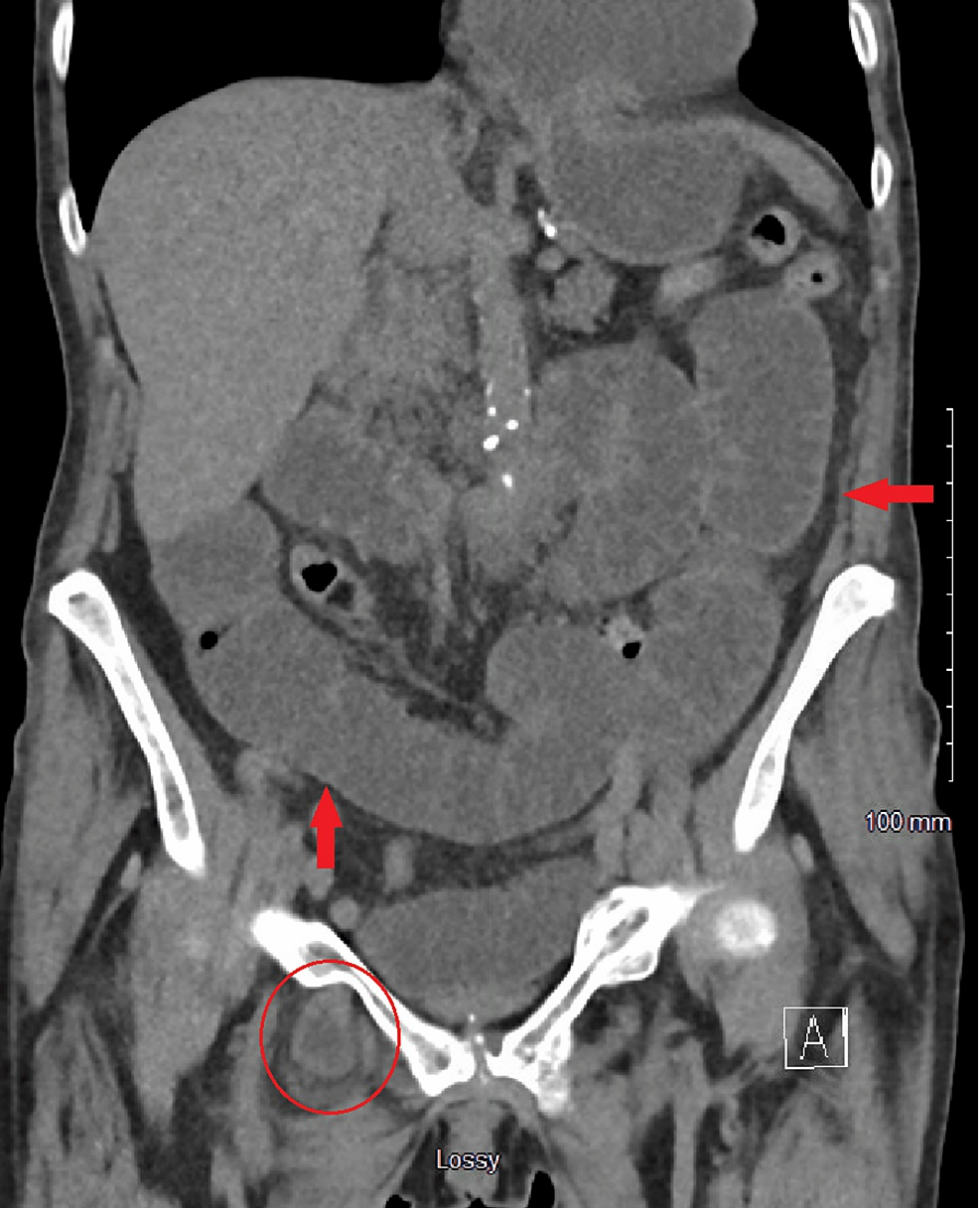
Coronal computed tomography on initial presentation in 2018 with incarcerated right obturator hernia (red circle) and dilated small bowel loops (red arrows), suggestive of small bowel obstruction.

**Figure 2:**
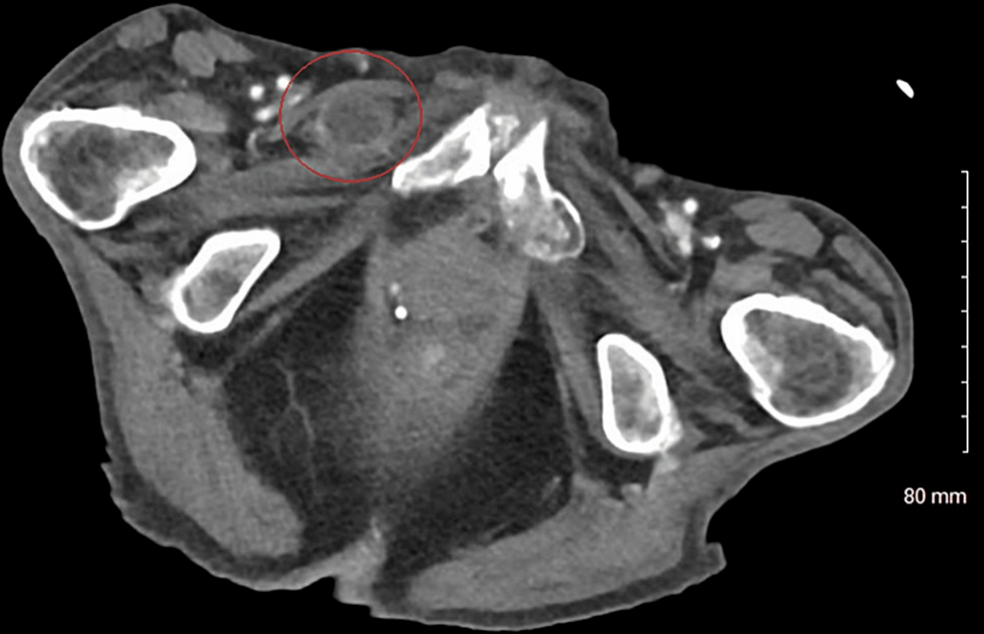
Axial computed tomography of our patient in 2022 demonstrating a recurrent right obturator hernia containing a piece of incarcerated small bowel (red circle).

**Figure 3:**
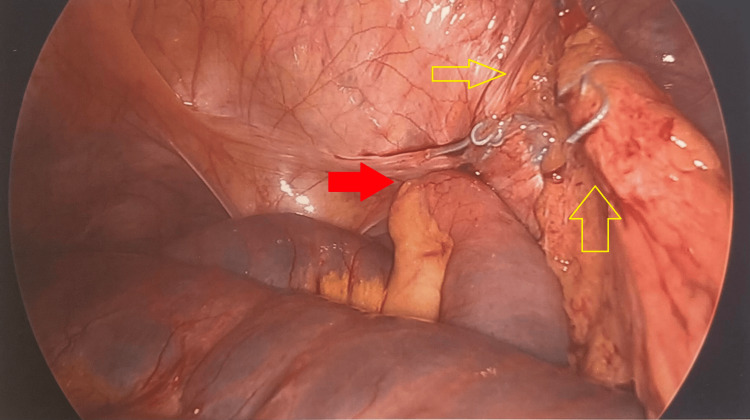
Diagnostic laparoscopy during patient presentation in 2022 noting a recurrent right obturator hernia containing a loop of small bowel (red arrow) and previous omental herniorrhaphy (yellow arrows).

**Figure 4:**
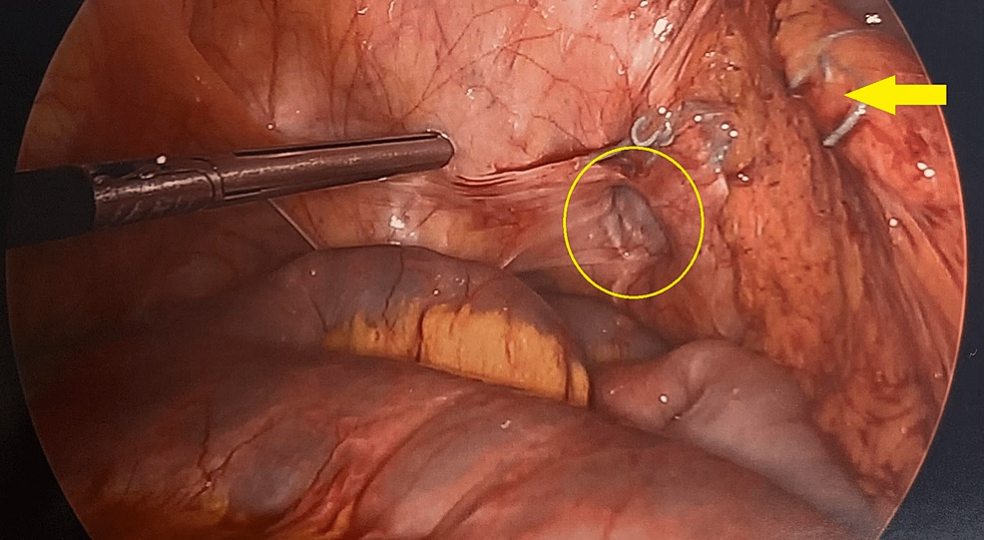
Intraoperative laparoscopic view of our patient’s right obturator hernia after bowel was reduced (yellow circle). Prior omental herniorrhaphy displaced to the right with previous Ethibond sutures (yellow arrow).

**Figure 5:**
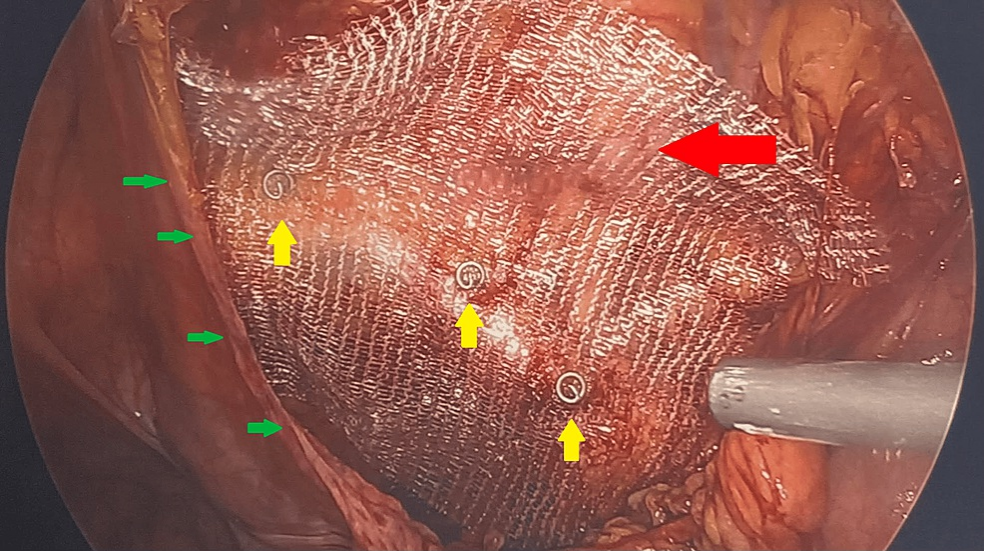
Mesh placement (red arrow) during TAPP repair with peritoneal flap (green arrows) and additional securement with titanium ProTacks (yellow arrows) along the pectineal ligament.

A 77-year-old female with a history significant for chronic kidney disease, chronic obstructive pulmonary disease, previous rectal cancer, and a body mass index (BMI) of 27 presented with right groin pain in 2018. She underwent CT showing an SBO due to a right obturator hernia (Figure 1) then promptly proceeded to the operating room (OR) for laparoscopic omental herniorrhaphy. A tongue of omentum was sutured to the peritoneum to patch the defect using 0 ethibond sutures. Her postoperative course was uncomplicated, and she was discharged on postoperative day 2 once tolerating a diet and having bowel function. She then re-presented four years later in 2022 with an SBO. Her BMI had decreased to 16 and was thought to be secondary to malnutrition from chronic disease. On CT, she was found to have a recurrent and incarcerated right obturator hernia containing a small bowel (Figure 2). She was taken to the OR for a diagnostic laparoscopy which revealed a recurrent right obturator hernia with a loop of small bowel in the right obturator foramen and displaced omental herniorrhaphy (Figures 3, 4). A laparoscopic TAPP repair with 8 x 9 cm Medtronic ProGrip^TM^ mesh was performed with additional securement to the pectineal ligament using Covidien titanium ProTacks^TM^ (Figure 5). She did well postoperatively and once tolerating a diet and having bowel function, was discharged postoperative day 2. She had a CT performed 14 months postoperatively for abdominal pain which showed no evidence of recurrence. 

## Discussion

Throughout the literature, there are few studies reporting obturator hernia repairs due to its low incidence. Some authors argue for open repair due to frailty or comorbidities of the affected patient population and inability to tolerate pneumoperitoneum. Furthermore, patients presenting with SBOs limits the amount of space for pneumoperitoneum and requires particular laparoscopic skills in the event that a small bowel resection is required. Others recommend laparoscopic repair which allows complete examination of the myopectineal orifice bilaterally to evaluate for any additional hernias such as inguinal or femoral. Holm et al performed a scoping review of 1299 patients with obturator hernias of which 937 (72%) underwent open repair and 295 (23%) underwent laparoscopic repair [4]. They reported a 10% recurrence rate with open primary repair, 2% recurrence with open mesh repair, and no documented recurrence with laparoscopic repair [4]. A review of 11 patients by Park reported only suture primary repair via the open approach in 7 and laparoscopic approach in 4 patients with no reported recurrence and an average follow up of 24 months (range 1-89 months) [2]. Given the increase in laparoscopic and robotic surgery, strong consideration should be made for performing minimally invasive pre-peritoneal mesh repairs on these patients when hemodynamics allow. As these patients are often frail and malnourished, the most durable repair with mesh should be performed to prevent recurrence in addition to complete evaluation for any additional pelvic hernias. We do not recommend repairing obturator hernias with omental herniorrhaphy as, unlike omental repair of perforated gastric or duodenal ulcers, there is minimal to no inflammatory response in these hernias to induce scarring and the prevention of future bowel incarceration. This, in addition to poor nutrition, is likely why our patient's hernia recurred in such a short amount of time. Regardless of repair technique, care should be taken to avoid injury to the obturator neurovascular bundle that traverses the obturator foramen antero-superiorly, or the corona mortis that runs nearby the foramen, thus the recommendation for avoiding dissection in the obturator foramen and placing an overlying piece of mesh.

## Conclusions

Despite the rare entity of obturator hernias, the basic tenets of a pelvic hernia repair applies. We recommend they be treated in a similar fashion, with reduction of herniated contents and a pre-peritoneal mesh repair for optimal durability if no contraindications exist. If the patient is a laparoscopic candidate, performing the repair using minimally invasive techniques is ideal to promote shorter recovery time, reduce hospitalization duration, and lessen narcotic usage. Otherwise, an open approach can be considered if the patient has existing conditions that precludes them from tolerating induced pneumoperitoneum.
